# Measuring mental well-being in Norway: validation of the Warwick-Edinburgh Mental Well-being Scale (WEMWBS)

**DOI:** 10.1186/s12888-017-1343-x

**Published:** 2017-05-12

**Authors:** Otto R. F. Smith, Daniele E. Alves, Marit Knapstad, Ellen Haug, Leif E. Aarø

**Affiliations:** 1 Department of Health Promotion, Norwegian Institute of Public Health, PO Box 973 Sentrum, N-5808 Bergen, Norway; 2 Department of Psychosocial Science, University of Bergen, Bergen, Norway; 3 Work Research Institute, Oslo and Akershus University of Applied Sciences, Oslo, Norway; 4 Department of Clinical Psycology, University of Bergen, Bergen, Norway; 5 Department of Health Promotion and Development, Faculty of Psychology, University of Bergen, Bergen, Norway; 6 NLA University College, Bergen, Norway

**Keywords:** Mental well-being, mental health, public health, measurement

## Abstract

**Background:**

Mental well-being is an important, yet understudied, area of research, partly due to lack of appropriate population-based measures. The Warwick-Edinburgh Mental Well-being Scale (WEMWBS) was developed to meet the needs for such a measure. This article assesses the psychometric properties of the Norwegian version of the WEMWBS, and its short-version (SWEMWBS) among a sample of primary health care patients who participated in the evaluation of Prompt Mental Health Care (PMHC), a novel Norwegian mental health care program aimed to increase access to treatment for anxiety and depression.

**Methods:**

Forward and back-translations were conducted, and 1168 patients filled out an electronic survey including the WEMWBS, and other mental health scales. The original dataset was randomly divided into a training sample (≈70%) and a validation sample (≈30%). Parallel analysis and confirmatory factor analysis were carried out to assess construct validity and precision. The final models were cross-validated in the validation sample by specifying a model with fixed parameters based on the estimates from the trainings set. Criterion validity and measurement invariance of the (S)WEMWBS were examined as well.

**Results:**

Support was found for the single factor hypothesis in both scales, but similar to previous studies, only after a number of residuals were allowed to correlate (WEMWBS: CFI = 0.99; RMSEA = 0.06, SWEMWBS: CFI = .99; RMSEA = 0.06). Further analyses showed that the correlated residuals did not alter the meaning of the underlying construct and did not substantially affect the associations with other variables. Precision was high for both versions of the WEMWBS (>.80), and scalar measurement invariance was obtained for gender and age group. The final measurement models displayed adequate fit statistics in the validation sample as well. Correlations with other mental health scales were largely in line with expectations. No statistically significant differences were found in mean latent (S)WEMWBS scores for age and gender.

**Conclusion:**

Both WEMWBS scales appear to be valid and precise instruments to measure mental well-being in primary health care patients. The results encourage the use of mental well-being as an outcome in future epidemiological, clinical, and evaluation studies, and may as such be valuable for both research and public health practice.

## Background

As declared by the World Health Organization (WHO), mental health is not solely characterized by the lack of negative symptoms or the absence of mental disorders [1]. Increasingly, the definition of mental health also incorporates the presence of psychological resources, encompassing both hedonic (subjective well-being) and eudemonic (psychological functioning) aspects [2, 3]. The WHO has even declared positive mental health to be the foundation for well-being and effective functioning for both the individual and the community [1]. The positive approach towards mental health has been applied in numerous areas [4]. For example in clinical psychology, it has been shown that interventions focusing on strengths and positive emotions can be as effective in treating mental disorders as more traditional approaches like cognitive behavioral therapy [5, 6]. Another example is the increased focus on positive attributes (assests) of people and/or communities within the area of public health [7]. Despite these advances, the field of positive mental health continuous to be under-researched partly because of the lack of appropriate population-based measures [8].

The Warwick-Edinburgh Mental Health Scale (WEMWBS) was developed to meet the need for a psychometrically sound measure of positive mental health [9]. The scale was derived from the “Affectometer 2”, a mental well-being scale with several favorable psychometric properties, but also with important limitations with regard to social desirability bias, item redundancy, and scale length [10]. Based on literature, validation results of the Affectometer 2, and input from focus groups, an expert panel agreed on key concepts and items that should be part of the new and improved scale. The key concepts were “positive affect and psychological functioning” (including autonomy, competence, self-acceptance, and personal growth), and “interpersonal relationships”. The final scale consisted of 14 positively worded items [9].

In the UK, the WEMWBS has been considered as an appropriate tool to measure mental well-being in different samples, such as overall population samples [9, 11, 12], students [9, 13], teenagers [14, 15], clinical samples [16, 17], and ethnic minority samples [18]. Yet, high values for Cronbach’s alpha led to the suspicion that item redundancy could be an issue for the WEMWBS as well. As a result, the 7-item short WEMWBS (SWEMWBS) was developed [19, 20]. The SWEMWBS has been preferred in terms of its psychometric properties and its convenience for monitoring positive mental health. However, it presents a more restricted definition of mental well-being as it mainly encompasses hedonic items. The WEMWBS may therefore be preferred when content coverage is an issue [19, 20]. It should also be noted that a recent study indicated limited discriminant validity of the WEMWBS when compared to the General Health Questionnaire (GHQ-12), a common mental distress measure [21].

To date the WEMWBS has been translated to more than a dozen languages such as Hindi, Urdu and Arabic [22]. Some of these translations have been validated and published, including Dutch, Italian, Spanish, and Portuguese versions [11, 23–26]. The Chinese, Norwegian, and Swedish version of the SWEMWBS have also been validated and published [17, 27]. The full version of the WEMWBS has not been validated in Norway yet and the validation of the Norwegian version of the SWEMWBS by Haver et al. [27] was conducted among Norwegian hotel managers only. It is therefore necessary to assess whether their findings can be generalized to other populations as well.

Several of the previous validation studies of the (S)WEMWBS (this abbreviation includes both WEMWBS and SWEMWBS) found evidence against the original proposed 1-factor structure [9, 13, 18, 27] as indicated by poor model fit. This may suggest multidimensionality, but a meaningful additional factor has not been identified so far [18]. Some studies improved model fit by including a number of correlated error terms to the 1-factor model [9, 27]. However, a clear justification, other than improving model fit, was not given. As models with and without correlated error terms may be differentially associated with relevant other variables, it would be of interest to examine the impact of correlated error terms in more detail.

An important advantage of the WEMWBS has been its combined brief and rich description of positive mental health, which is useful in monitoring mental health in the population, as well as in evaluating mental health programs. One such program in Norway is Prompt Mental Health Care (PMHC), which is modelled after the English program Improving Access to Psychological Therapies (IAPT) [28]. Like PMHC, IAPT is a free-of-charge, low-threshold, primary health care program, aimed at reaching adults with anxiety and mild to moderate levels of depression. Cognitive behavioural therapy (CBT) is provided by multidisciplinary teams of health care professionals including at least one psychologist. PMHC was launched in Norway in 2012 and has to date been expanded to 23 sites across the country [29].

As a mental health program, PMHC aims to reduce symptoms of depression and anxiety, as well as to increase work participation, quality of life and mental well-being. The WEMWBS has been used to measure the latter outcome. The aim of the present study is to examine the psychometric properties of the original and the abbreviated WEMWBS in this Norwegian sample of primary care patients.

## Methods

### Participants

Eighteen hundred and 58 patients received treatment at PMHC between October 2014 and February 2016 across 14 different sites. Of the 1858 patients that received treatment, 1189 participated in the study, resulting in an overall participation rate of 64%. Participation was based on opt-in, where all eligible clients where invited after the initial assessment by a PMHC therapist. All participants provided written informed consent upon recruitment. Patients either were referred to the service by their general practitioners or contacted the free-of-charge service themselves. Eligible patients were adults with anxiety and/or low to moderate levels of depression, and whose home address was within their respective PMHC site. Patients with suspected psychosis, bipolar disorder, personality disorder, severe drug abuse, and suicide risk were generally excluded from PMHC, and were referred to the GP or more specialized mental health care services. The data material presented in this manuscript is based on the responses to a set of questionnaires that all participating PMHC patients completed prior to treatment. The study was approved by the Regional Committee for Medical Research Ethics in Norway (nr. 2014/597).

### Measures

#### The Warwick- Edinburgh mental well-being scale (WEMWBS)

The WEMWBS is a 5-point Likert scale consisting of 14 items, which can be ranged from “none of the time” to “all of the time”. A global score was calculated by adding up item scores, ranging from 14 to 70. The higher the global score, the higher the level of mental well-being. The original WEMWBS showed high reliability, low social desirability bias, and confirmatory factor analysis supported the single-factor hypothesis, after allowing some of the residuals to correlate [9]. Moreover, the original scale showed high positive correlations with other well-being scales, and low to moderate positive correlation with overall health [9].

The SWEMWBS consist of 7 items, and was found to have good psychometric properties as well [20, 24]. Haver et al. [27] assessed the validity of the SWEMBS among Norwegian and Swedish hotel managers, and reported acceptable psychometric properties.

#### Translation into Norwegian

The method of forward and back translation was used to translate the original scale to Norwegian, as advised by Health Scotland [30]. In Stage 1, the original scale was independently translated by an expert panel of four people, of whom two were native Norwegian speakers. All four were fluent in both English and Norwegian. Three had knowledge about the instrument. In stage 2, the translators agreed upon a synthesized version with a recording observer present. In stage 3, the synthesized version was translated back to the original language, English, by two additional independent translators with fluency in both English and Norwegian. In stage 4, the expert panel developed a pre-final version of the questionnaire for field testing. Finally, in stage 5 a small sample of psychology students at the University of Bergen (*N* = 5) completed the pre-final version of the questionnaire, and were assessed for question comprehension and interpretation. As this did not lead to further changes, the pre-final version was adopted as the final version of the Norwegian WEMWBS.

#### Other measures

Other measures were used to assess criterion validity of WEMBWS. All measures included in this study were self-administered.

The Patient Health Questionnaire (PHQ-9) was used to measure depressive symptoms [31]. It included 9 items based on each of the DSM-IV criteria for depression, and could range from 0 (“none of the time) to 3 (“all of the time”). This yielded a total sum score that ranged from 0 to 27. Cronbach’s alpha was .85 in the current sample.

The Generalized Anxiety Disorder Assessment (GAD-7) was used to measure anxiety [32]. It includes 7 items to score common anxiety symptoms, and contains the same response alternatives as the PHQ-9, ranging from 0 (“none of the time) to 3 (“all of the time”). Total score could range from 0 to 21. In addition to measuring generalized anxiety disorder, there are indications that the GAD-7 also has good sensitivity and specificity for panic, social anxiety, and post-traumatic stress disorder [32]. Cronbach’s alpha was .87 in the current sample.

The EQ-5D-5 L was used to measure functional health status [33]. It included 5 items measuring mobility, self-care, usual activity, pain/discomfort, and anxiety/depression, and could range from 1 (no problem) to 5 (extreme problem/unable to carry out activity). This yielded a total sum score that ranged from 5 to 25. The higher the score, the *lower* the level of functional status. Cronbach’s alpha was .66 in the current sample.

An abbreviated version of the Mindful Attention Awareness Scale (MAAS-5) was used to measure awareness of and attention to whatever is happening in the present [34–36]. It included 5 items ranging from 1 to 6 from which a mean can be computed. Higher scores reflect higher levels of mindfulness. Cronbach’s alpha was .87 in the current sample.

The Brief Self-Control Scale (BSCS) is a short version of the Self-Control Scale which measures five domains: Controlling thoughts, controlling emotions, controlling impulses, regulating behavior/performance, and habit-breaking [37]. It included 13 items, and the Norwegian version could range from 1 (Disagree Very Strongly) to 6 (Agree very strongly) for 5 items, and 1 (never) to 6 (always) for the remaining 8 items. Higher scores reflect *lower* levels of self-control. Cronbach’s alpha was .77 in the current sample.

### Statistical analyses

Prior to analyses, the original dataset (*N* = 1168) was divided into a training sample (≈70%) and a validation sample (≈30%) by means of a random split [38]. The training set was used for all analyses described below, while the validation set was only used to validate the final measurement models.

Item level descriptive statistics were calculated to examine the distributional properties of the WEMWBS.

For each WEMWBS item, a univariate ordinal probit variance component model was fitted in order to examine whether it was necessary to account for the cluster effects of pilot site (average cluster size = 64). Intraclass correlation coefficients were calculated for each item as the proportion of the residual between-group variance and the total variance (probit variance at the within-group level was standardized to 1). The largest ICC’s were found for item 13 (ICC = .009) and item 4 (ICC = .013). The ICC’s of all other items were ≤.002. Given the very small ICC’s across all items, accounting for the cluster effect of pilot site was deemed unnecessary.

A parallel analysis was carried out to determine whether a multidimensional factor structure was supported in our data based on the eigenvalues from the sample correlation matrix. For this particular analysis, we treated the 5-point Likert scale of the WEMWBS as continuous. The 95th percentile criterion was used to decide on the number of factors. Results indicated that only the first eigenvalue of the sample correlation matrix (λ_1_ = 6.45; λ_2_ = 1.19) was larger compared to the 95th percentile eigenvalues (λ_1p_ = 1.27; λ_2p_ = 1.21) of the parallel analysis, which supported the 1-factor structure of the WEMWBS.

Confirmatory factor analysis based on the polychoric correlation matrix was fitted to the data, which provides statistical results analogous to Samejima’s Graded Response Model [39]. This model was used to assess the unidimensional fit and the construct validity of both the full Norwegian WEMWBS and SWEMWBS (indicators specified as categorical, robust weighted least squares estimator (WLSMV), parameterization Theta). The mean and variance of the latent factor were set to respectively 0 and 1. WLSMV allows for partially missing data. Only 1.2% of the participants did not complete any of the WEMWBS items, whereas the percentage of missingness per item varied between .9% and 2.5%. The Root Mean Square Error of Approximation (RMSEA) and the Comparative Fit Index (CFI) were used as goodness of fit measures. An RMSEA close to or lower than .06 and a CFI close to or higher than .95 was adopted to indicate good model fit [40]. The initial 1-factor models (WEMWBS and SWEMWBS) were specified without correlated errors. If model fit was poor, correlated errors were added in a stepwise fashion based on the largest standardized expected parameter change (SEPC) until adequate fit statistics were obtained [41], similar to the procedure adopted by Tennant et al. [9]. The final models were cross-validated in the validation sample by specifying a model with fixed parameters based on the estimates obtained from the trainings set.

Measurement invariance was examined separately for gender (male vs female), and equal sized age groups (18–30; 31–43; ≥44). First, configural invariance was tested by estimating the 1-factor model of the WEMWBS in each group without constraining factor loadings and intercepts. In the next step, metric invariance was tested by constraining the factor loadings to be equal in each group. Due to the categorical nature of the indicators, additional constraints were required to test metric invariance; the first threshold of each item was held equal across groups and the second threshold of the item that was used to set the metric of the factor was also held equal across groups [42]. Finally, both factor loadings and thresholds were constrained to be equal across groups to test for scalar invariance. Scalar invariance is required for comparing absolute scores across groups. In many cases, full scalar invariance cannot be obtained and one or more of the constrained model parameters need to be set free in order to improve model fit. According to recommendations from Byrne et al. [43] partial scalar invariance is obtained when at least two of the factor indicators are invariant. Testing strict measurement invariance, in which residual variances are fixed to one across groups, was considered less relevant for the present study since correlated errors were explicitly accounted for in the measurement model [44]. Adjustments to the model were informed by SEPC. In line with Cheung and Rensvold [45], we used a change in CFI of more than 0.01 as an indicator of true difference in relative model fit.

Conditional precision across the WEMWBS construct was assessed by utilizing the information function with values rescaled between 0 and 1 (Rescaled conditional precision = 1–1/Information). Values close to 1 indicate high conditional precision. Cronbach’s alpha was also calculated. Criterion validity was assessed by calculating Pearson correlations between the WEMWBS scores and the PHQ-9, GAD-7, EQ-5D-5 L, MAAS-5, and BSCS-13. We expected high correlations (|r| > .5) with depressive and anxious symptoms [20], moderate correlations (.3 < |r| < .5) with functional health status/overall health [9, 13], and low correlations (|r| < .3) with the MAAS-5 and the BSCS-13 [27, 34].

To examine the impact of taking into account measurement error and correlated error terms on structural parameter estimates, the correlations with the criterion variables were calculated for latent (S)WEMWBS scores with and without correlated errors, and manifest (S)WEMWBS scores. This resulted in three correlations per WEMWBS version for each criterion variable. To express the relative difference between these three estimates, relative bias (|r|-|r_ref_|/|r_ref_|) was calculated using the correlations between the criterion variables and the latent scores from the (S)WEMWBS model with correlated errors as the reference. Small relative bias for the estimated structural correlations based on the latent (S)WEMWBS model without error terms as compared to the model with error terms would support the justification of the latter model. Not accounting for measurement error typically attenuates the size of the correlation between variables and lower reliability would therefore result in larger relative bias when applying manifest sum scores. Finally, mean WEMWBS scores across age and gender were examined. We expected similar levels of mental well-being across age groups, and men to have higher levels of mental well-being as compared to women [9].

The Statistical Package for Social Science (SPSS) version 22 was used to prepare the data file and for basic descriptive statistics. Mplus version 7.11 was used for all other analyses.

## Results

### Sample and item characteristics

The training sample included 799 participants of which 73% were women, 44% had higher education than secondary school, and 61% were living with a partner or spouse. Mean age was 37.3 (SD = 12.6). Forty percent were employed, 34% were on sick leave, 5% were receiving disability pension, and 21% belonged to another occupational status category.

As displayed in Table 1, all categories were used and there were at least 6 responses for each answer category across all items. There was evidence for skewness for some of the items, in particular items 3, 5, 10 and 13, while kurtosis was most pronounced for items 9 and 12. In histograms, the WEMWBS and SWEMWBS sum scores seemed normally distributed, and there were no indications for floor and/or ceiling effects. The mean manifest sum scores of the WEMWBS and the SWEMWBS were 38.6 (SD = 8.9) and 20.0 (SD = 4.5).

**Table 1:** Item level descriptive statistics

WEMWBS item	None of the time, % (*n*)	Rarely, % (*n*)	Some of the time, % (*n*)	Often, % (*n*)	All of the time, % (*n*)	Mean (SD)	Skewness (z-score)	Kurtosis (z-score)
1. I’ve been feeling optimistic about the future	8.9 (69)	31.8 (248)	39.7 (309)	17.6 (137)	2.1 (16)	2.72 (.92)	0.69	- 2.27
2. I’ve been feeling useful	7.4 (58)	31.9 (249)	41.3 (326)	17.4 (136)	1.4 (11)	2.73 (.88)	0.17	- 2.10
3. I’ve been feeling relaxed	10.7 (83)	43.4 (338)	35.6 (277)	9.3 (72)	1.0 (8)	2.47 (.84)	3.38	- 0.29
4. I’ve been feeling interested in other people	3.6 (28)	21.3 (167)	39.9 (313)	28.1 (220)	7.1 (56)	3.14 (.95)	−0.34	- 2.29
5. I’ve had energy to spare	29.3 (230)	42.1 (331)	22.0 (173)	5.9 (46)	0.8 (6)	2.07 (.90)	6.90	- 0.36
6. I’ve been dealing with problems well	7.3 (57)	26.4 (207)	48.3 (378)	16.3 (128)	1.7 (13)	2.79 (.86)	- 1.22	- 0.43
7. I’ve been thinking clearly	4.6 (36)	18.7 (147)	47.5 (373)	23.4 (184)	5.7 (45)	3.07 (.91)	- 0.55	- 0.01
8. I’ve been feeling good about myself	16.2 (127)	37.6 (295)	34.6 (271)	10.2 (80)	1.4 (11)	2.43 (.93)	2.82	- 1.89
9. I’ve been feeling close to other people	6.0 (47)	22.2 (175)	35.6 (280)	28.8 (227)	7.4 (58)	3.09 (1.02)	- 1.25	- 3.10
10. I’ve been feeling confident	19.6 (154)	41.1 (323)	27.4 (215)	10.1 (79)	1.8 (14)	2.33 (.96)	5.30	- 1.28
11. I’ve been able to make up my own mind about things	3.9 (31)	19.2 (151)	43.4 (341)	24.6 (193)	8.9 (70)	3.15 (.96)	0.28	- 0.15
12. I’ve been feeling loved	7.3 (57)	19.0 (149)	29.9 (235)	27.4 (215)	16.5 (130)	3.27 (1.16)	- 2.07	- 4.53
13. I’ve been interested in new things	18.8 (147)	34.5 (270)	28.8 (225)	13.8 (108)	4.1 (32)	2.50 (1.07)	4.39	- 2.84
14. I’ve been feeling cheerful	3.3 (26)	29.5 (231)	51.3 (402)	14.4 (113)	1.5 (12)	2.81 (.77)	1.17	0.87

### Construct validity

Confirmatory factor analysis was conducted to test the hypothesized one-factor structure of the Norwegian version of WEMBWS, and goodness of fit for the single-factor model was tested. The initial model, assuming no dependencies among residuals, showed poor fit (CFI = 0.90; RMSEA = 0.11). After adding correlated error terms in a stepwise fashion, adequate fit statistics were obtained after 10 steps (CFI = 0.99; RMSEA = 0.06), as seen in Table 2. The final model was tested in the validation sample and displayed adequate fit statistics (CFI = 0.99; RMSEA = 0.03).

**Table 2:** Unstandardized parameter estimates for items of the WEMWBS and SWEMWBS, and model fit estimates for four different one-factor models: WEMWBS models with and without correlated error terms, and SWEMWBS models with without correlated error terms

Item number	14-item version, no correlated errors	14-item version with correlated errors^a^	7-item version, no correlated errors	7-item version with correlated errors^b^
1	0.97	1.04	0.91	0.87
2	1.14	1.26	1.16	1.16
3	0.65	0.61	0.58	0.62
4	0.75	0.65		
5	0.85	0.86		
6	1.09	0.96	1.33	1.07
7	0.98	0.83	1.19	0.82
8	1.69	1.47		
9	0.94	0.77	0.72	0.87
10	1.37	1.19		
11	0.94	0.87	1.05	0.84
12	0.72	0.60		
13	0.96	0.99		
14	1.07	1.11		
Chi-square (df)	12,094.06 (91)	246.66 (67)	233.22 (14)	30.28 (9)
RMSEA	0.14	0.06	0.14	0.06
CFI	0.90	0.99	0.95	1.00

^a)^ Correlated errors in order of inclusion: i9-i12, i8-i10, i4-i9, i6-i7, i7-i11, i11-i6, i5-i3, i14-i3, i13-i4, i12-i4.

^b)^ Correlated errors in order of inclusion: i1-i2, i9-i6, i6-i7, i11-i7, i11-i6.

The same analyses were conducted to test the hypothesized one-factor structure of the Norwegian short-version of the scale, the SWEMWBS. Like the 14-item model, confirmatory factor analysis of the 7-item model, assuming no dependencies among residuals, showed relatively poor fit (CFI = 0.95; RMSEA = 0.11). Adequate model fit (CFI = .99; RMSEA = 0.06) was obtained after adding 5 correlated error terms. As can be seen in Table 2, adding correlated error terms had relatively little effect on the discrimination parameters (factor loadings). The final model of the SWEMWBS was tested in the validation sample and displayed adequate fit statistics as well (CFI = 0.98; RMSEA = 0.04). A value .95 was found for the correlation between manifest scores of the WEMWBS and SWEMWBS.

### Measurement invariance

The configural model for the WEMWBS with correlated errors yielded an acceptable fit for both gender (RMSEA = .06; CFI = .98) and age group (RMSEA = .07; CFI = .98). Subsequent estimation of the metric and scalar models yielded acceptable model fit statistics as well. For gender, ΔCFI was <.001 for the metric vs configural comparison and .001 for the scalar vs metric comparison. For age, ΔCFI was .004 for the metric vs configural comparison and .002 for the scalar vs metric comparison. Similar results were obtained when testing the measurement invariance of the SWEMWBS.

### Precision

**Fig. 1:**
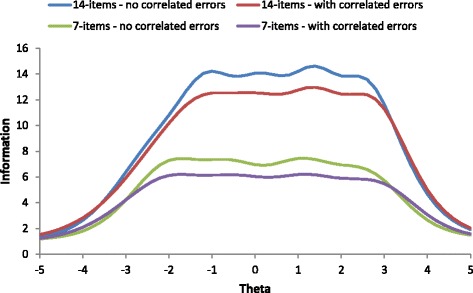
Information curves of the (S)WEMWBS with and without correlated error terms

As displayed in Fig. 1, the conditional precision for the WEMWBS was >.90 ± 2SD from the mean for both the initial model without correlated errors and the final model with correlated errors. For the SWEMWBS, the conditional precision was >.80 for ±2SD from the mean for the models with and without correlated errors. For comparison, Cronbach’s alpha was .91 and .83 for the WEMWBS and the SWEMWBS, respectively. It should be noted that the average inter-item correlation for the WEMWBS was .42 (18.7% of inter-item correlations >.5, range = .21 to .73), whereas the average inter-item correlation for the SWEMWBS was .41 (23.8% of inter-item correlations >.5, range = .24 to .61). The lower alpha for the SWEMWBS seems therefore primarily the result of fewer items, and not per se due to the removal of redundant items.

### Criterion validity

The correlation of the mental well-being scores with depressive symptoms varied between -.67 and −.62, in line with expectations (see Table 3). Moderate correlations were found with anxiety (−.46 < *r* < −.46) and functional health status (−.47 < *r* < −.44). The correlation with mindfulness was low (.27 < *r* < .29), whereas the correlation with self-control was somewhat higher than expected (−.37 < *r* < −.35). Still, this correlation was significantly lower as compared to the correlations with anxiety and functional status. Relative bias appeared to be small in most cases, except for manifest SWEMWBS scores. For three out of five correlations with manifest SWEMWBS, the relative bias exceeded 10% (Table 3).

**Table 3:** Criterion validity of the (S)WEMWBS scale^a^

	PHQ-9	GAD-7	EQ-5D-5 L	MAAS-5	BSCS-13
Latent WEMWBS with correlated errors^b^	−.71 (−.75, −.67)	−.48 (−.54, −.43)	−.49 (−.55, −.44)	.30 (.23, .37)	−.38 (−.44, −.31)
Latent WEMWBS without correlated errors	−.68 (−.72, −.64)	−.47 (−.52, −.41)	−.47 (−.53, −.42)	.29 (.23, .36)	−.37 (−.43, −.30)
Manifest WEMWBS	−.67 (−.71, −.63)	−.46 (−.51, −.40)	−.45 (−.51, −.40)	.29 (.21, .36)	−.36 (−.43, −.28)
Latent SWEMWBS with correlated errors^c^	−.72 (−.76, −.67)	−.51 (−.57, −.45)	−.50 (−.55, −.44)	.32 (.25, .39)	−.41 (−.47, −.35)
Latent SWEMWBS without correlated errors	−.68 (−.72, −.63)	−.48 (−.54, −.43)	−.48 (−.53, −.42)	.31 (.24, .38)	−.40 (−.46, −.33)
Manifest SWEMWBS	−.64 (−.68, −.60)	−.47 (−.53, −.41)	−.44 (−.49, −.38)	.29 (.22, .37)	−.36 (−.43, −.29)
Relative bias^d^: Latent WEMWBS, no corr. Errors	−.04	−.02	−.04	−.03	−.03
Relative bias^d^: Manifest WEMWBS	−.06	−.04	−.08	−.03	−.05
Relative bias^d^: Latent SWEMWBS, no corr. Errors	−.06	−.06	−.04	−.03	−.02
Relative bias^d^: Manifest SWEMWBS	−.11	−.08	−.12	−.09	−.12

^a^ First 6 rows display bivariate correlations and the 95% confidence intervals between parentheses. The remaining 4 rows display estimates for relative bias. ^b)^ Reference for relative bias WEMWBS. ^c)^ Reference for relative bias SWEMWBS. ^d)^ (|r|-|r_ref_|)/|r_ref_|

No statistically significant age differences were found for latent (S)WEMWBS with correlated error terms (WEMWBS: middle-aged adults vs young adults, *b* = −.02, *p* = .78; older adults vs young adults, *b* = .04, *p* = .65; SWEMWBS: middle-aged adults vs young adults, *b* = .03, *p* = .70; older adults vs young adults, *b* = .08, *p* = .24). The observed mean differences between women and men were also not statistically significant (WEMWBS: *b* = −09, *p* = .24; SWEMWBS: *b* = −.04, *p* = .55). Similar results were obtained for latent (S)WEMWBS without correlated error terms and manifest (S)WEMWBS scores.

## Discussion

There has been an increased interest for measuring positive aspects of mental health [46]. The WEMWBS was developed in the UK as a broad measure of mental well-being capturing both hedonic and eudemonic aspects with good psychometric properties. The primary aim of this study was to validate the WEMWBS and its abbreviated version (SWEMWBS) in a sample of Norwegian primary health care patients who suffer from anxiety and/or mild-to-moderate depression.

The unidimensional nature of the WEMWBS was confirmed by means of a parallel analysis, and CFA indicated acceptable model fit after including a number of correlated error terms. The latter is generally considered bad praxis, as correlated error terms should only be added in case these can theoretically be justified [44]. Correlated error terms can alter the meaning of the underlying latent construct, and can bias structural parameter estimates. However, in the case of the WEMWBS, our results showed that the relative bias in structural parameter estimates of associations with relevant criterion variables were small for the (S)WEMWBS with and without correlated error terms, and manifest WEMWBS scores. As expected, the relative bias was somewhat higher for manifest SWEMWBS scores as 3 out of 5 correlations had relative biases exceeding 10%. Forero et al. [47] labeled a relative bias of more than 10% as “substantial”, but a general consensus on what is substantial bias seems to be lacking. Nonetheless, our finding suggests that relative bias should be taken into the equation when using manifest SWEMWBS scores.

In contrast to some previous studies [19, 20], the present study did not formally test whether the SWEMWBS fitted the Rasch model. However, given that it did not fit the less restrictive graded response model, it would be safe to conclude that the Rasch model did not hold in this sample of Norwegian primary health care patients. Future studies should examine whether it’s possible to derive an abbreviated Norwegian version of the WEMWBS that fulfills the criteria of the Rasch model, which ultimately would result in equal interval scaling of WEMWBS scores, and would facilitate valid examination of change scores [9, 19, 20, 24].

No evidence was found for floor or ceiling effects, suggesting the scale has potential to examine change during the course of treatment. In addition, the precision estimates of both WEMWBS versions were good. Full scalar measurement invariance was obtained for gender and age group, suggesting that meaningful comparisons of the WEMWBS scores across these groups can be made. Previous studies found measurement invariance across age [15], but not across gender [15, 19]. Associations with criterion variables were largely as expected, and in line with previous findings [9, 11, 15, 19, 27].

### Limitations

A number of limitations should be mentioned. The sample consists of primary health care patients only, and the findings may therefore not be extended to the general Norwegian population. Moreover, responsiveness of the Norwegian versions of WEMWBS and SWEMWBS should be assessed in future studies in order to address whether they are responsive to change, as was the original scale [48]. The current study provided limited information on the discriminant validity of the WEMWBS. In the light of recent findings [21], more research is needed to determine how different the WEMWBS is from other competing measures. Finally, test-retest reliability and measurement invariance across time were not tested.

## Conclusion

In summary, both the full and short WEMWBS scales appear to be valid and precise instruments to measure well-being in Norwegian primary health care patients with anxiety and/or mild-to-moderate depression. The SWEMWBS has a clear advantage over the WEMWBS due to its brevity. Future studies are warranted to confirm these findings in similar and other populations, and extend the validation work as outlined in the limitations section. The results of the present study encourage the use of mental well-being as an outcome in epidemiological, intervention and evaluation studies, and may as such be valuable for both research and public health practice.

## Abbreviations

BSCS: Brief self-control scale
CFI: Comparative fit index
EQ-5D-5 L: EuroQol – 5 dimensions – 5 levels
GAD: Generalized anxiety disorder
IAPT: Improving access to psychological therapies
MAAS: Mindful attention awareness scale.
PHQ: Patient health questionnaire
PMHC: Prompt mental health care
RMSEA: Root mean square error of approximation
SEPC: Standardized expected parameter change
SWEMWBS: Short Warwick-Edinburgh mental health scale
(S)WEMWBS: Short & original Warwick-Edinburgh mental health scale
WEMWBS: Warwick-Edinburgh mental health scale
WHO: World Health Organization
WLSMV: Weighted least squares mean and variance-adjusted

## References

[1] WHO (2004). Promoting Mental Health; Concepts emerging evidence and practice. Summary report.

[2] Ryan RM, Deci EL (2001). On happiness and human potentials: A review of research on hedonic and eudaimonic well-being. Annu Rev Psychol.

[3] Diener E (1984). Subjective well-being. Psychol Bull.

[4] Linley PA, Joseph S (2004). Positive psychology in practice.

[5] Seligman ME, Steen TA, Park N, Peterson C (2005). Positive psychology progress: empirical validation of interventions. Am Psychol.

[6] Sin NL, Lyubomirsky S (2009). Enhancing well-being and alleviating depressive symptoms with positive psychology interventions: a practice-friendly meta-analysis. J Clin Psychol.

[7] Morgan A, Ziglio E (2007). Revitalising the evidence base for public health: an assets model. Promot Educ.

[8] Hu Y, Stewart-Brown S, Twigg L, Weich S (2007). Can the 12-item General Health Questionnaire be used to measure positive mental health?. Psychol Med.

[9] Tennant R, Hiller L, Fishwick R, Platt S, Joseph S, Weich S, Parkinson J, Secker J, Stewart-Brown S (2007). The Warwick-Edinburgh Mental Well-being Scale (WEMWBS): development and UK validation. Health Qual Life Outcomes.

[10] Tennant R, Joseph S, Stewart-Brown S (2007). The Affectometer 2: a measure of positive mental health in UK populations. Qual Life Res.

[11] Castellví P, Forero C, Codony M, Vilagut G, Brugulat P, Medina A, Gabilondo A, Mompart A, Colom J, Tresserras R (2014). The Spanish version of the Warwick-Edinburgh Mental Well-Being Scale (WEMWBS) is valid for use in the general population. Qual Life Res.

[12] Lloyd K, Devine P (2012). Psychometric Properties of the Warwick–Edinburgh mental well-being scale (WEMWBS) in Northern Ireland. J Ment Health.

[13] López M, Gabilondo A, Codony M, García-Forero C, Vilagut G, Castellví P, Ferrer M, Alonso J (2013). Adaptation into Spanish of the Warwick-Edinburgh Mental Well-being Scale (WEMWBS) and preliminary validation in a student sample. Qual Life Res.

[14] Clarke A, Friede T, Putz R, Ashdown J, Martin S, Blake A, Adi Y, Parkinson J, Flynn P, Platt S (2011). Warwick-Edinburgh Mental Well-being Scale (WEMWBS): validated for teenage school students in England and Scotland. A mixed methods assessment. BMC Public Health.

[15] Hunter SC, Houghton S, Wood L (2015). Positive Mental Well-Being in Australian Adolescents: Evaluating the Warwick-Edinburgh Mental Well-Being Scale. Aust Educ Dev Psychol.

[16] Crawford MJ, Robotham D, Thana L, Patterson S, Weaver T, Barber R, Wykes T, Rose D (2011). Selecting outcome measures in mental health: the views of service users. J Ment Health.

[17] Ng SS, Lo AW, Leung TK, Chan FS, Wong AT, Lam RW, Tsang DK (2014). Translation and validation of the Chinese version of the short Warwick-Edinburgh Mental Well-being Scale for patients with mental illness in Hong Kong. East Asian Arch Psychiatr.

[18] Taggart F, Friede T, Weich S, Clarke A, Johnson M, Stewart-Brown S: Cross cultural evaluation of the Warwick- Edinburgh mental well-being scale (WEMWBS) -a mixed methods study. Health Qual. Life Outcomes2013, 11(1):1-12. 10.1186/1477-7525-11-27 PMC3610169 23445544

[19] Stewart-Brown S, Tennant A, Tennant R, Platt S, Parkinson J, Weich S (2009). Internal construct validity of the Warwick-Edinburgh Mental Well-being Scale (WEMWBS): a Rasch analysis using data from the Scottish Health Education Population Survey. Health Qual Life Outcomes.

[20] Bartram D, Sinclair J, Baldwin D (2013). Further validation of the Warwick-Edinburgh Mental Well-being Scale (WEMWBS) in the UK veterinary profession: Rasch analysis. Qual Life Res.

[21] Bohnke JR, Croudace TJ (2016). Calibrating well-being, quality of life and common mental disorder items: psychometric epidemiology in public mental health research. Br J Psychiatry.

[22] WEMWBS in other languages**.** [http://www2.warwick.ac.uk/fac/med/research/platform/wemwbs/researchers/languages/].

[23] Gremigni P, Stewart-Brown S (2011). Measuring mental well-being: Italian validation of the Warwick-Edinburgh Mental Well-being Scale (WEMWBS). G Ital Psicol.

[24] Stewart-Brown SL, Platt S, Tennant A, Maheswaran H, Parkinson J, Weich S, Tennant R, Taggart F, Clarke A (2011). The Warwick-Edinburgh Mental Well-being Scale (WEMWBS): a valid and reliable tool for measuring mental well-being in diverse populations and projects. J Epidemiol Community Health.

[25] JJAd S, TAd C, Guilherme JH, WCd S, LRL A, Krebs JA, Sotoriva P (2015). Adaptation and cross-cultural validation of the Brazilian version of the Warwick-Edinburgh mental well-being scale. Rev. Assoc. Med. Bras.

[26] Waqas A, Ahmad W, Haddad M, Taggart FM, Muhammad Z, Bukhari MH, Sami SA, Batool SM, Najeeb F, Hanif A, et al. Measuring the well-being of health care professionals in the Punjab: a psychometric evaluation of the Warwick-Edinburgh Mental Well-being Scale in a Pakistani population. Peerj. 2015;3:–e1264. 10.7717/peerj.1264 PMC4636413 26557420

[27] Haver A, Akerjordet K, Caputi P, Furunes T, Magee C (2015). Measuring mental well-being: A validation of the Short Warwick-Edinburgh Mental Well-Being Scale in Norwegian and Swedish. Scand J Public Health.

[28] Clark DM, Layard R, Smithies R, Richards DA, Suckling R, Wright B (2009). Improving access to psychological therapy: Initial evaluation of two UK demonstration sites. Behav Res Ther.

[29] Smith OR, Alves DE, Knapstad M (2016). Rask Psykisk Helsehjelp: Evaluering av de første 12 pilotene i Norge.

[30] Measuring mental well-being. [http://www.healthscotland.com/scotlands-health/population/Measuring-positive-mental-health.aspx].

[31] Kroenke K, Spitzer RL, Williams JB (2001). The PHQ-9: validity of a brief depression severity measure. J Gen Intern Med.

[32] Spitzer RL, Kroenke K, Williams JB, Lowe B (2006). A brief measure for assessing generalized anxiety disorder: the GAD-7. Arch Intern Med.

[33] Nord E, Johansen R (2015). Transforming EQ-5D utilities for use in cost–value analysis of health programs. Eur J Health Econ.

[34] Brown KW, Ryan RM (2003). The benefits of being present: mindfulness and its role in psychological well-being. J Pers Soc Psychol.

[35] Osman A, Lamis DA, Bagge CL, Freedenthal S, Barnes SM (2016). The Mindful Attention Awareness Scale: Further Examination of Dimensionality, Reliability, and Concurrent Validity Estimates. J Pers Assess.

[36] Smith OR, Melkevik O, Samdal O, Larsen TM, Haug E. Psychometric properties of the five-item version of the Mindful Awareness Attention Scale (MAAS) in Norwegian adolescents. Scand J Public Health. 2017;1403494817699321 10.1177/1403494817699321 28382831

[37] Tangney JP, Baumeister RF, Boone AL (2004). High self-control predicts good adjustment, less pathology, better grades, and interpersonal success. J Pers.

[38] Davenport TH, McNeill D. Analytics in Healthcare and the Life Sciences: Strategies, Implementation Methods, and Best Practices. Upper Saddle River: Pearson FT Press; 2013.

[39] McDonald RP (1999). Test theory: A unified approach.

[40] Hu L, Bentler PM (1999). Cutoff criteria for fit indexes in covariance structure analysis: Conventional criteria versus new alternatives. Struct Equ Model Multidiscip J.

[41] Whittaker TA (2012). Using the Modification Index and Standardized Expected Parameter Change for Model Modification. J Exp Educ.

[42] Millsap RE (2011). Statistical approaches to measurement invariance.

[43] Byrne BM, Shavelson RJ, Muthén B (1989). Testing for the equivalence of factor covariance and mean structures: The issue of partial measurement invariance. Psychol Bull.

[44] Wu AD, Li Z, Zumbo BD. Decoding the meaning of factorial invariance and updating the practice of multi-group confirmatory factor analysis: a demonstration with TIMSS data. Pract Assess Res Eval. 2007;12

[45] Cheung GW, Rensvold RB (2002). Evaluating goodness-of-fit indexes for testing measurement invariance. Struct Equ Model.

[46] Proctor C, Tweed R, Morris D (2015). The Naturally Emerging Structure of Well-Being Among Young Adults: 'Big Two' or Other Framework?. J Happiness Stud.

[47] Forero CG, Maydeu-Olivares A, Gallardo-Pujol D (2009). Factor Analysis with Ordinal Indicators: A Monte Carlo Study Comparing DWLS and ULS Estimation. Struct Equ Model Multidiscip J.

[48] Maheswaran H, Weich S, Powell J, Stewart-Brown S (2012). Evaluating the responsiveness of the Warwick Edinburgh Mental Well-Being Scale (WEMWBS): Group and individual level analysis. Health Qual. Life Outcomes.

